# Announcement

**Published:** 1890-01

**Authors:** F. W. McRae

**Affiliations:** Atlanta, Ga.


### Editorial.

### ANNOUNCEMENT.

The readers of the JOURNAL will observe that with this issue my sole proprietorship ceases. Dr. L. P. Kennedy and Dr. M. B. Hutchins having each purchased a third interest in the JOURNAL, it will in the future be conducted under our joint proprietorship and management. Dr. Kennedy will have entire charge of the subscription department, and Dr. Hutchins of the advertising department. Both are men of marked ability and high professional attainments.

Dr. Kennedy is a graduate of Erskin College, South Carolina. He received his medical degree from the Medical Department of the University of New York, class of 1887. After graduating he accepted a position in the hospitals on Blackwell's Island for six months, and then spent nine months in Charity Hospital, Brooklyn.

Dr. Hutchins graduated from the Atlanta Medical College, class of 1886, with the highest honor, and was valedictorian of his class. After spending a year and a half in general practice, he decided to devote himself exclusively to diseases of the skin. He has just returned from New York, where he has been since March, 1888, eighteen months of which time were spent in the New York Skin and Cancer Hospital.

We take great pleasure in announcing the connection of these gentlemen with the JOURNAL, for we feel assured their association will prove of much benefit to its friends and patrons.

No change will be made in the editorial staff.

THE JOURNAL was never in a more prosperous condition. The actual circulation of this issue is five thousand copies, which though confined chiefly to the South, includes nearly every State and territory in the Union.

Arrangements have been perfected for getting original articles from some of the best writers North and South.

Our regular correspondents will furnish our readers the gist of what is going on in the hospitals and medical societies of the larger cities.

Our translators will furnish the JOURNAL with selections from the latest and best French and German literature.

Our items will concisely mirror current medical thought.

We will spare neither money nor labor where the JOURNAL'S interests demand them, and will be satisfied with nothing less than the best medical journal in the South.

F. W. MCRAE.

*Atlanta, Ga., December 23, 1889.*

